# Osmolyte-producing microbial biostimulants regulate the growth of *Arachis hypogaea* L. under drought stress

**DOI:** 10.1186/s12866-024-03320-6

**Published:** 2024-05-15

**Authors:** Sakthi Uma Devi Eswaran, Lalitha Sundaram, Kahkashan Perveen, Najat A. Bukhari, R. Z. Sayyed

**Affiliations:** 1 Soil Biology and PGPR Lab, Department of Botany, Periyar University, Salem, 636011 India; 2 Department of Botany & Microbiology, College of Science, King Saud University, P.O. Box-22452, Riyadh, 11495 Saudi Arabia; 3 Department of Microbiology, PSGVP Mandal’s S I Arts, G B Patel Science and STKV Sangh Commerce College, Shahada, 425409 India

**Keywords:** Antioxidants, Exopolysaccharide, Microbial biostimulants, Osmotic stress, PGP traits

## Abstract

Globally, drought stress poses a significant threat to crop productivity. Improving the drought tolerance of crops with microbial biostimulants is a sustainable strategy to meet a growing population’s demands. This research aimed to elucidate microbial biostimulants’ (Plant Growth Promoting Rhizobacteria) role in alleviating drought stress in oil-seed crops. In total, 15 bacterial isolates were selected for drought tolerance and screened for plant growth-promoting (PGP) attributes like phosphate solubilization and production of indole-3-acetic acid, siderophore, hydrogen cyanide, ammonia, and exopolysaccharide. This research describes two PGPR strains: *Acinetobacter calcoaceticus* AC06 and *Bacillus amyloliquefaciens* BA01. The present study demonstrated that these strains (AC06 and BA01) produced abundant osmolytes under osmotic stress, including proline (2.21 and 1.75 µg ml^− 1^), salicylic acid (18.59 and 14.21 µg ml^− 1^), trehalose (28.35 and 22.74 µg mg^− 1^ FW) and glycine betaine (11.35 and 7.74 mg g^− 1^) respectively. AC06 and BA01 strains were further evaluated for their multifunctional performance by inoculating in *Arachis hypogaea* L. (Groundnut) under mild and severe drought regimes (60 and 40% Field Capacity). Inoculation with microbial biostimulants displayed distinct osmotic-adjustment abilities of the groundnut, such as growth parameters, plant biomass, photosynthetic pigments, relative water content, proline, and soluble sugar in respective to control during drought. On the other hand, plant sensitivity indexes such as electrolyte leakage and malondialdehyde (MDA) contents were decreased as well as cooperatively conferred plant drought tolerance by induced alterations in stress indicators such as catalase (CAT), ascorbate peroxidase (APX), and superoxide dismutase (SOD). Thus, *Acinetobacter* sp. AC06 and *Bacillus* sp. BA01 can be considered as osmolyte producing microbial biostimulants to simultaneously induce osmotic tolerance and metabolic changes in groundnuts under drought stress.

**Supplementary Information:**

The online version contains supplementary material available at 10.1186/s12866-024-03320-6.

## Introduction

In recent decades, environmental climate changes and adverse abiotic factors have significantly impaired crop productivity and agricultural yields worldwide [1]. Drought is a severe abiotic factor constraining plants and the human population due to decreased precipitation worldwide in the 21st century. Drought severity, water scarcity, and extreme temperatures alter soil moisture, affecting plant survival and crop productivity [2, 3]. This stress results in stunted plant growth, lowers crop yield, hampers molecular metabolism, increases cellular reactive oxygen species (ROS), and affects morphological, physiological, and biochemical attributes that negatively impact plant health growth and crop productivity [4, 5]. Under drought, perceiving these stress signals, plants are triggered to respond through events such as osmolyte accumulation, ROS scavenging, and production of functional proteins [6–8]. However, natural drought stress responses enacted by plants are insufficient to survive under severe drought.

Peanut or Groundnut (*Arachis hypogaea* L.) is a leguminous, macrothermal, oleaginous crop cultivated in tropical and subtropical regions of the world with a substantial economic value for India, China, Nigeria, and the United States [9, 10]. As a rain-fed crop, groundnut is more prone to periodic drought stress, devastatingly affecting its quality and distribution. Globally, according to recent estimations, groundnut productivity faces an annual loss of approximately 6 million tons owing to drought [11, 12]. Extensive research has been dedicated to establishing traditional breeding techniques, developing drought-tolerant transgenic plants through genetic engineering, shifting crop cultivation, and undertaking resource management practices as strategies to cope with drought. Unfortunately, these strategies are very complex, laborious, time-consuming, even loss of favorable host plant characteristics, and also neglect the role of the microbiome [4, 13, 14]. Consequently, we need substantial and reliable in situ strategies to nourish plants under constrained irrigations.

The recruitment of microbial biostimulants is suggested as an intelligent strategy to mitigate abiotic stress [15]. Plant growth-promoting rhizobacteria (PGPR) or microbial biostimulants form a subgroup of the heterogeneous family of biostimulants that have captured the attention of the agriculture industry [16]. These microbial biostimulants are fertilizing products that stimulate plant nutrition processes to improve high nutrient utilization and increase tolerance to abiotic stress and crop quality [17]. PGPR as biostimulants are most beneficial for agricultural sustainability by promoting and safeguarding the overall plant growth and yield directly or indirectly under severe drought stress [18–23].

PGPR are the natural predominant habitants, co-evolved with rhizosphere soil over millennia, that rely on root exudates to fuel their metabolic activities [24]. PGPR is a crucial biological component with favorable effects on plant biochemical and physiological properties under drought conditions [25–27]. PGPR are known to improve plant growth and its survival in drought-stressed environments through mechanisms such as phytohormones production, phosphate solubilization, exopolysaccharide, and siderophore production for sequestration of iron [28, 29]. These rhizobacteria are osmotolerant and alter the roots’ hormonal balance, resulting in microbial colonization and symbiotic relationships under osmotic stress [30].

The osmotic stress tolerance of PGPR is due to their osmolyte production potential. Drought triggers PGPR to produce compatible osmolytes such as proline, trehalose, sucrose, glycine betaine, and ectoine [31, 32]. It has been documented that osmolytes accumulating PGPR alleviate plant osmotic stress [33, 34]. Microbial biostimulants should promise to facilitate sustainable agriculture and could be of particular interest to overcoming twin pressures of water scarcity and increasing population under future climate change scenarios. Therefore, the present investigation focused on (i) isolating and screening osmotolerant microbial biostimulants, (ii) evaluating its potential for osmolyte productions, and (iii) analyzing the morphological, physiological, and biochemical responses of groundnut upon biostimulant inoculation under drought stress. Practically, we hypothesized that osmolyte synthesizing PGPR biostimulants could help groundnut grow under stress conditions and strengthen the host–partner (PGPR) relationship, thus producing highly drought-resilient plants.

## Materials and methods

### Source of rhizobacteria

Healthy plant samples of *Arachis hypogaea* L. were uprooted from the agricultural field of Salem district (11º86ʹN, 78º07ʹ E), Tamil Nadu, India, in March 2022. The area is considered an arid/semi-arid region with an average rainfall of 232 mm and an average humidity of 6.8% at sampling. The following are the physicochemical properties of the soil: pH 7.2, EC 0.32 dS m^−1^, organic matter 2.16%, available nitrogen 56.2 mg kg^−1^, available phosphorus 17.8 mg kg^−1,^ and potassium 217 mg kg^−1^. The rhizospheric soil was removed aseptically and serially diluted, and the appropriate dilutions were spread onto tryptic soy agar (TSA)supplemented with ascending levels of PEG 6000 (0–25%) to ensure different osmotic potentials (0 to − 0.73 MPa) [35]. The plates were incubated at 30ºC for 24–48 h. The bacterial colonies grown with distinct morphology were considered osmotolerant, serially purified, and conserved as glycerol stocks at -80ºC for further studies.

### In vitro bioassay for multifarious PGP traits

Drought-tolerant rhizobacteria were characterized in vitro for direct plant growth-promoting properties like indole acetic acid (IAA), siderophore and phosphate solubilization, and indirect multifunctional traits such as hydrogen cyanide (HCN), and ammonia production, as described earlier [23]. Briefly, auxin production as IAA was determined by the inoculation of bacterial culture (10^8^ CFU/ml) in nutrient broth (NB) amended with L-tryptophan (100 µg ml^− 1^). The positive reaction in the grown culture was identified as the color change by adding 4 ml of Salkowski’s reagent followed by absorbance recording at 530 nm. Phosphate solubilization was measured qualitatively using Pikovskaya’s agar medium containing 0.5% tricalcium phosphate. The phosphate solubilization index (PSI) was determined as follows –

$$PSI= \frac{Colony \ diameter+Halo \ zone \ diameter}{colony \ diameter}$$

The inoculated plates were incubated for 72 h at 28 ± 2ºC. The onset of the solubilization zone around the bacterial colonies was observed for positive indication. Qualitative and quantitative siderophore production was assessed by spot inoculation of rhizobacteria on (Chrome Azurol S) CAS-agar plates pursued by the occurrence of orange halo zone and 1 ml cell-free supernatant to 1 ml of CAS reagent examined spectrometrically at 630 nm, respectively. HCN production was determined by the color change of overlaid filter paper presaturated with 0.5% picric acid and 2% sodium carbonate solution upon culturing the bacteria in NA supplemented with 0.4% glycine. The presence of ammonia was identified by the development of yellow color in the inoculated peptone broth after adding 0.5 ml of Nessler’s reagent.

### Assessment of rhizobacterial growth at varied osmotic pressures

Different osmotic pressures (− 0.05, − 0.30, − 0.49, − 0.73, and − 1.03 MPa) were prepared by adding different concentrations of PEG 6000 (5, 10, 15, 20, 25, and 30%) to NB inoculated with 1% exponentially grown (10^8^ CFU/ml) rhizobacterial suspension [36]. Following the incubation for 72 h at 30ºC, the drought-tolerant potential of the strains was evaluated in terms of minimum inhibitory concentration (MIC) by measuring the optical density at 600 nm [37].

#### Exopolysaccharide (EPS) production assay

Extraction and quantitative estimation of EPS by the bacterial isolates were performed in a Basal medium in the presence of -1.03 MPa (30% PEG) and the absence of osmotic stress. After inoculation, the medium was incubated at 30 ± 2ºC for 5–8 days. Centrifugation at 10,000 rpm for 10 min was done to extract the pellet. The cell-free supernatant was suspended with double the volume of ice-cold isopropanol. The solution was incubated overnight at 4ºC. The extracted EPS was centrifuged, washed, dried at 40ºC for 24 h, and weighed [38].

#### Biochemical characterization and genotyping of selected rhizobacteria

The selected drought-tolerant isolates were subsequently examined for their preliminary morphological and biochemical characteristics, such as Gram staining, oxidase, catalase, starch hydrolysis, and IMViC (Indole, Methyl red, Voges-Proskauer, and Citrate utilization) tests employing standard protocols of Cappuccino and Sherman [39]. The isolates were genetically identified by partial 16S rRNA gene sequence. Genomic DNA was extracted, and the quality was analyzed on 0.8% agarose gel electrophoresis. The universal 16S rRNA gene region was amplified by PCR using universal primers 27F (5’-AGAGTTTGATCMTGGCTCAG-3’) and 1492R (5’-TACGGYTACCTTGTTACGACTT-3’) according to Giongo et al. [40]. The amplified PCR product of 16 S rRNA genes was purified using a GeneJET PCR purification kit (Thermo Scientific, EU-Lithuania). The final purified PCR products were sequenced using the Big Dye Terminator 3.1 sequencing kit (Applied Biosystems, USA). The homology searches were analyzed manually by BLASTn (Basic Local Alignment Search Tool) and compared against the sequence available in GenBank from the National Centre for Biotechnology Information (NCBI). Phylogenetic analysis of 16 S rRNA sequences with the reference sequences was done and aligned using Molecular Evolutionary Genetic Analysis (MEGA software version 11) [41]. The phylogenetic tree was constructed using the Neighbor-Joining method with bootstrapping analysis of 1000 replicates. The 16 S rRNA sequence of *Acinetobacter johnsonii*, *A. tjernbergiae*, and *A.tandoii* was used to assign an outgroup for *A. calcoaceticus*, and *Bacillus tequilensis* was used as an outgroup for *B. amyloliquefaciens*. The output sequences were submitted in the NCBI GenBank database with the accession numbers.

### Oxidative stress features of drought-tolerant microbial biostimulant

#### Proline

Proline production by the drought-tolerant microbial biostimulant was estimated as described by Abou-Aly et al. [42]. The PGPR isolates were cultured in NB with different osmotic potentials of PEG 6000 (0 and 30%), incubated at 28ºC for 24 h, and centrifuged (10,000xg) to obtain supernatant. To 2 ml of bacterial supernatant, 2 ml of acid ninhydrin (2.5 g ninhydrin in 60 ml glacial acetic acid and 40 ml of 6 M phosphoric acid), and 2 ml of glacial acetic acid was added, followed by incubation for 1 h in a boiling water bath, cooled and 4 ml of toluene was added for extraction under vigorous mixing for 15–20 s. The chromophore containing toluene was separated and used to measure the absorbance at 520 nm using toluene as a blank.

#### Salicylic acid

The capacity of the drought-tolerant rhizobacteria to produce salicylic acid (SA) was estimated following the protocol of Abou-Aly et al. [42] as follows: 4 ml of rhizobacterial supernatant (cultured in NB amended with 0 and 30% PEG) was acidified with 1 N HCl to reach pH 2. Salicylic acid was extracted in chloroform (CHCl_3_) 1:1, and then 4 ml of distilled water and 5 ml of 2 M FeCl_3_ were added. The aqueous phase containing purple iron-SA complex was used to read absorbance at 527 nm, using aqueous phase without chromophore as blank.

#### Trehalose quantification

The accumulation of trehalose was determined by the modified protocol of García et al. [33]. Briefly, strains were grown to a 10^8^ CFU/ml bacterial density corresponding to 0.5 OD units for 24 h at 30ºC in NB supplemented with various concentrations of PEG (0 and 30%). The bacterial cultures with density of 10^8^ CFU/ml were centrifuged, washed with water, and resuspended in 80% ethanol. After incubation at 85ºC for 15 min, the mixture was centrifuged at 10,000 x g for 5 min, and the supernatant was recovered. The samples were air-dried to remove excess ethanol and resuspended in sterile deionized water. Trehalose concentration was analyzed by HPLC using column Aminex HPX-87 C (Bio-rad Labs, Richmond, CA, United States) with acetonitrile and water (80:20 v/v). Pure trehalose was employed to determine the standard peak and values.

#### Glycine betaine production

The methods of Vasconcellos et al. [43] determined the glycine betaine quantification by incubating the isolates in NB with varying concentrations of PEG (0 and 30%) at 27ºC for 24 h. The bacterial cells were pelletized by centrifugation (5000 x g), weighted, and resuspended in 1 ml of ethanol by vigorous shaking (30 min). The extract was then filtered through a 0.45 μm filter and evaluated for glycine betaine production by injecting in HPLC on an RP-18 column (Merck, Germany) and detected at 200 nm.

### Plant growth promotion under drought stress - Pot experiment

The pot study determined the effect of PGPR-based microbial biostimulants on alleviating the behavior of groundnuts under drought stress during the planting season of groundnuts from November–December (2022). Details about the materials and techniques adopted during experimentation, plant sampling, and analyses were described here.

#### Plant material and inoculum preparation

An *in-situ* pot culture study was conducted in the greenhouse with a rain exclusion roof, Department of Botany, Periyar University (11º43’09"N, 78º04’36.5"E) Salem, Tamil Nadu, India. Seeds of *Arachis hypogaea* L. were procured from Tamil Nadu Agricultural University, Coimbatore, Tamil Nadu, India. The seeds were surface-sterilized by soaking them in 1% sodium hypochlorite solution for 20 min and washing them thrice with distilled water.

Application of PGPR microbial biostimulant was given as soil drench at the time of sowing and 14 days after the first inoculation. AC06 and BA01 strains were selected for vitro osmotic stress tolerance. For trials, rhizobacteria from the preserved stock were cultured in nutrient broth aerobically on a rotary shaker (150 rpm) for 48 h at 28 ± 2ºC. The bacterial suspension was adjusted to a final concentration of 10^8^CFU/ml, having an optical density of 0.5 at 600 nm by spectrophotometer. The resulting suspension was used as inoculums (10 ml per pot) and uninoculated medium as control.

#### Experimental design and execution

The sterilized seeds (8 per pot) were implanted in a pot (25 × 19 cm) filled with sieved, air-dried, and uniformly mixed sandy-clay loam soil (3:1). The main characteristics of the soil are presented in Table 1.

**Table 1:** Physicochemical analysis of soil used in pot experiment

Soil analysis		Value
Mechanical		
Texture		Sandy Loam
Particle size distribution	Sand (%)	63.89
	Slit (%)	15.74
	Clay (%)	20.08
**Chemical**		
pH		7.54
EC (dS m^− 1^)		1.64
Organic Matter (%)		3.16
Organic carbon (%)		5.7
Available P (mg kg^− 1^)		15.41
Available K (mg kg^− 1^)		208
Available N (mg kg^− 1^)		53.67

The experiment was laid out in a randomized complete block design with the factorial arrangement under non-sterile conditions with three replicates. Following the emergence, we thinned eight seedlings to five healthy and uniform plants per pot. The factorial components include six combined levels of inoculation, without inoculation as control, and two levels of drought stress. Subsequently, the following nine experimental trials were performed:

T1 – Control with 100% Field Capacity (FC).

T2 – Inoculation with AC06.

T3 – Inoculation with BA01.

T4 –Mild Drought (MD) (60% FC).

T5 – Inoculation with AC06 under MD.

T6 – Inoculation with BA01 under MD.

T7 – Severe Drought (SD) (40% FC).

T8 – Inoculation with AC06 under SD.

T9 – Inoculation with BA01 under SD.

#### Irrigation and drought stress imposition

The seeds were grown under an entire irrigation regime for two weeks. Afterward, irrigation was halted for all seedlings to initiate the drought stress. The drought stress was induced by withholding water (suppression of water). At the 4-leaf stage (2 weeks after emergence), the seedlings were exposed to water stress by limiting irrigation levels to respective field capacities of 100 (without drought), 60 (mild drought), and 40% (severe drought). The Field Capacity (FC) of the pot soil was calculated by using the equation:

$$FC= \frac{water \ added\left(ml\right)-water \ leached\left(ml\right)in \ 24 \ h}{soil \ weight \left(g\right)}\times 100$$

The plants were grown with a photoperiod of 16 h light/8 h dark cycle, the light intensity of 1000µmolm^− 2^s^− 1^, temperature of 22 ± 2ºC/20ºC, and relative humidity of ∼ 65% for 30 days. At the end of the experimentation, plants were harvested for the phenotypical and biochemical indices.

#### Measurements of plant morphological traits

Agronomic attributes like plant shoot and root length were estimated with a ruler. The plant’s fresh weight was weighed using analytical balance. Samples were oven-dried at 80ºC to achieve constant dry weight.

### Measurements of plant physiological characters

#### Relative water content (RWC)

The relative water content was determined according to the standard method of Barrs and Weatherly [44] with some modifications. Five leaves were harvested, their fresh weight was immediately noted, and they were immersed in distilled water. The turgor weights of the fully saturated leaves were analyzed after soaking for 24 h. The leaves were dehydrated in the oven at 70ºC for 48 h to measure dry weight. RCW was computed as follows:

$$RWC \left(\%\right)=\frac{Fresh \ weight-Dry \ weight}{Turgor \ weight-Dry \ weight}\times 100\%$$

#### Electrolyte leakage (EL)

The methodology of Danish et al. [45] was used to evaluate the leakage of electrolytic ions from the leaves. In 20 ml deionized water, about 1 g leaf samples were immersed and incubated at 25ºC for 24 h. The initial conductivity (EC1) was measured using a pre-calibrated electrical conductivity (EC) meter. The test tube was heated at 120ºC for 20 min in a water bath and the final EC (EC2). EL was quantified as follows:

$$Electrolyte \ leakage \left(EL\%\right)=\frac{EC1}{EC2}\times 100$$

#### Membrane stability index (MSI)

The treated plants’ MSI was measured using a conductivity (EC) meter [46]. For the assay, 200 mg leaf samples were placed in two glass vials containing 10 ml distilled water. One set was heated at 40ºC for 30 min in a water bath, and electrical conductivity (C1) was measured. The second set was boiled at 100ºC for 10 min, and final conductivity (C2) was measured. MSI was estimated as follows:

$$MSI=\left[1-\left(\frac{C1}{C2}\right)\right]\times 100$$

#### Assessment of greenness index and photosynthetic pigments

The greenness index of the intact leaves of groundnut was examined using a portable SPAD – 502 chlorophyll meter (Konica Minolta, Tokyo, Japan), which measures ‘in situ’ without damaging leaves. The measurements were taken between 9:00 a.m. to 12:30 p.m. local time. Lichtenthaler [47] defined the method for estimating the chlorophyll and carotenoid content. Frozen leaf samples (0.5 g) were homogenized with 15 ml of 80% acetone, and the solution was centrifuged at 15,000 x g for 35 min. The absorbance of the supernatant was estimated using a UV-VIS spectrophotometer (Shimadzu UV-1700, Tokyo, Japan) at a wavelength of 663 nm, 645 nm, and 470 nm.

### Measurements of plant biochemical characters

#### Estimation of proline content

Proline content was assayed as described previously [48] with slight modifications. One gram of fresh leaves was homogenized with 6 ml of 5% sulphosalicyclic acid. Subsequently, the extract was centrifuged (10,000 x g) for 10 min, and 1 ml of resultant supernatant was made to 2 ml with distilled water. 2 ml ninhydrin was added to it and incubated for 45 min at 100ºC. The mixture was cooled and extracted with an equal volume of toluene. The toluene phase containing the pink chromophore was monitored at an optical density of 520 nm. Quantification of proline level was estimated by comparing the standard curve of L-proline.

#### Determination of lipid peroxidation

Lipid peroxidation, estimated as malondialdehyde (MDA) production, was quantified by the thiobarbituric acid (TBA) reaction, which follows the method of Heath and Packer [49] with a minor modification as proposed by Armendariz et al. [50]. Briefly, 200 mg fresh leaves were homogenized in 600 µl of 0.1% trichloroaceticacid (TCA). Centrifugation (15,000 x g) for 20 min at 4ºC was done to obtain supernatant. 1.5 ml of 20% TCA containing 0.5% TBA was mixed with 0.5 ml of supernatant. The mixture was heated in a water bath (95ºC, 25 min) and allowed to cool in an ice bath, followed by recentrifugation (15,000 x g, 4ºC, 5 min). The supernatant was read for absorbance at 532 nm. Expression of the calculated result was indicated in terms of µmol MDA g^− 1^ fresh weight.

#### Estimation of total soluble sugars (TSS)

Dubois et al. [51] method enumerated the TSS. Briefly, 0.5 g of fresh leaves was grounded with 80% ethanol or deionized water. After centrifugation, 2.5 ml of H_2_SO_4_ and 1 ml of phenol were added to 0.5 ml of supernatant, followed by incubation at 37ºC for 1 h. Then, optical density was recorded at 485 nm.

$$Sugar \left(\mu g/ml\right)=\frac{sample \ absorbance \times dilution \ factor\times K value}{weight of sample}$$

#### Inoculation response of microbial biostimulant on stress markers under drought

Plant stress markers or antioxidant enzymes such as catalase (CAT), ascorbate peroxidase (APX), and superoxide dismutase (SOD) were assessed following the methods of Chen et al. [52], Nakano and Asada [53] and Dhindsa et al. [54], respectively.

Fresh leaf samples (1 g) were homogenized and extracted in 1:10 with buffer solution 50 mM phosphate buffer (10 ml, pH 7) amended with 1% polyvinyl pyrrolidone (PVPP). The extract was centrifuged at 10,000 x g for 30 min at 4ºC. The supernatant was used as a crude enzyme for antioxidant bioassays.

Catalase (EC 1.11.1.6) was estimated by monitoring the deduction in absorbance of H_2_O_2_ at 240 nm per minute at every 10-second interval. The reaction mixture (3 ml) consisted of 100 µl enzyme extract, 1.9 ml of 50mM phosphate buffer (pH 7.0), and 1 ml of 0.3% H_2_O_2_. The activity of CAT was expressed as one unit with 0.01 differences in absorbance at 240 nm.

The oxidation rate of ascorbate at 290 nm monitored ascorbate peroxidase (EC 1.11.1.11) activity. A 1 ml reaction mixture contained 0.05 ml enzyme extract, 0.25 ml of 100mM potassium phosphate buffer (pH 7.0), 0.25 ml of 0.4 mM EDTA, 0.25 ml of 1 mM ascorbic acid, 0.01 ml of 10 mM H_2_O_2_ and 0.19 ml of distilled water. The subsequent decrease in ascorbic acid was observed at 0.01 absorbance min^− 1^.

A decrease in the absorbance due to the color formation by nitro-blue tetrazolium (NBT) determined superoxide dismutase (EC 1.15.1.1). A 100 µl of crude enzyme extract was added to the mixture of 0.2 ml of 200 μm methionine, 0.1 ml of EDTA (3mM), 0.1 ml of riboflavin (2 µM), 0.1 ml of NBT (2.25 mM), 1.5 ml of phosphate buffer (100 mM. pH 7.8) and 0.1 ml of sodium carbonate (1.5 M). The final volume (3 ml) was adjusted by distilled water and illuminated under light of 4000 flux for 10 min for the development of purple color formation. The absorbance was determined at 560 nm. One unit of SOD was considered as the amount of enzyme required for 50% inhibition of NBT reduction.

### Determination of rhizospheric bacterial population

The rhizospheric microbial population was recorded 30 days after inoculation. The rhizospheric soil samples were taken and transferred to the laboratory and stored at 4 °C. Total microbial counts were calculated using the serial dilution method described by Alexander and Zuberer [55]. The samples were inoculated in agar plates and incubated at 28 ± 2 °C for 2–3 days. The microbial populations were counted using the colony counter and expressed in terms of the log of colony-forming units (CFU) per gram of rhizospheric soil.

### Statistical analysis

All the biological measurements were performed in triplicate, and the standard deviation (SD) was calculated for all mean values. The data were statistically evaluated by one-way ANOVA (SPSS 20.0 version Japan Inc., Tokyo, Japan). Differences between treatments were considered significant at *p* ≤ 0.05 using Tukey’s *post-hoc* test for multiple comparisons. Figures were plotted using GraphPad Prism 5 software. SR plot was utilized to create Pearson’s correlation matrix for the variables under consideration.

## Results

The osmotolerant or drought-tolerant PGPR were isolated and characterized in tolerance to osmotic stress with the prospects of designing microbial biostimulants with improved strains. In total, 15 different bacterial isolates were isolated from arid soil. The isolates were selected based on their resistance to osmotic stress concentrations applied using PEG 6000. All isolates were able to grow up to an osmotic stress potential of -0.05 MPa; 70% of isolates showed tolerance at – 0.15 and – 0.30 MPa, while 30% of isolates have higher resistance to – 0.49 and – 0.73 MPa osmotic potential. Further, the rhizobacterial isolates were subjected to PGP characteristics.

### In vitro multifarious PGP traits associated with drought tolerant activity

From the results in Table 2, the isolated rhizobacterial isolates were screened for PGP properties of IAA, siderophore, phosphate solubilization, ammonia, and HCN. All the isolates produced IAA except SA02, ES13, ES14, BA06, and SB05. The highest value of IAA was tested for AC06 (128.82 µg ml^− 1^), followed by BA01 (97.31 µg ml^− 1^), SB14 (78.22 µg ml^− 1^), ES12 (70.42 µg ml^− 1^), and SA04 (63.06 µg ml^− 1^). However, the IAA production level for other isolates was lower (less than 60 µg ml^− 1^).

**Table 2:** Osmotic resistance and plant growth promoting traits of selected rhizobacterial isolates

Isolates	Osmotic resistance to PEG-6000	IAA (µg ml^− 1^)	Siderophore Units (%)	PSI	NH_3_	HCN
SAU1	− 0.15	13.80 ± 1.22	-	1.5	+	-
SA02	− 0.15	-	09.33 ± 0.01	2.9	+	+
SA03	− 0.05	21.98 ± 1.07	17.73 ± 0.69	-	+	-
SA04	− 0.49	63.06 ± 1.20	60.75 ± 0.11	4.2	-	+
AC05	− 0.05	39.73 ± 1.25	-	3.8	-	+
AC06	− 0.73	128.82 ± 1.37	84.87 ± 0.81	9.9	+	+
ES12	− 0.49	70.42 ± 0.94	62.16 ± 0.09	5.0	+	-
ES13	− 0.30	-	12.06 ± 0.45	-	+	-
ES14	− 0.05	-	10.43 ± 0.73	-	+	-
ES15	− 0.49	41.93 ± 1.84	-	4.2	-	-
BA01	− 0.73	97.31 ± 0.62	79.16 ± 0.94	7.5	+	+
BA06	− 0.05	-	22.84 ± 1.35	5.2	-	+
SB30	− 0.30	48.04 ± 1.30	-	2.7	-	-
SB14	− 0.49	78.22 ± 0.63	65.81 ± 0.72	5.3	+	-
SB05	− 0.30	-	20.64 ± 1.31	-	+	+

(+) Positive; (-) Negative and no activity found. Data is a mean of triplicate ± standard deviation

**Fig. 1:**
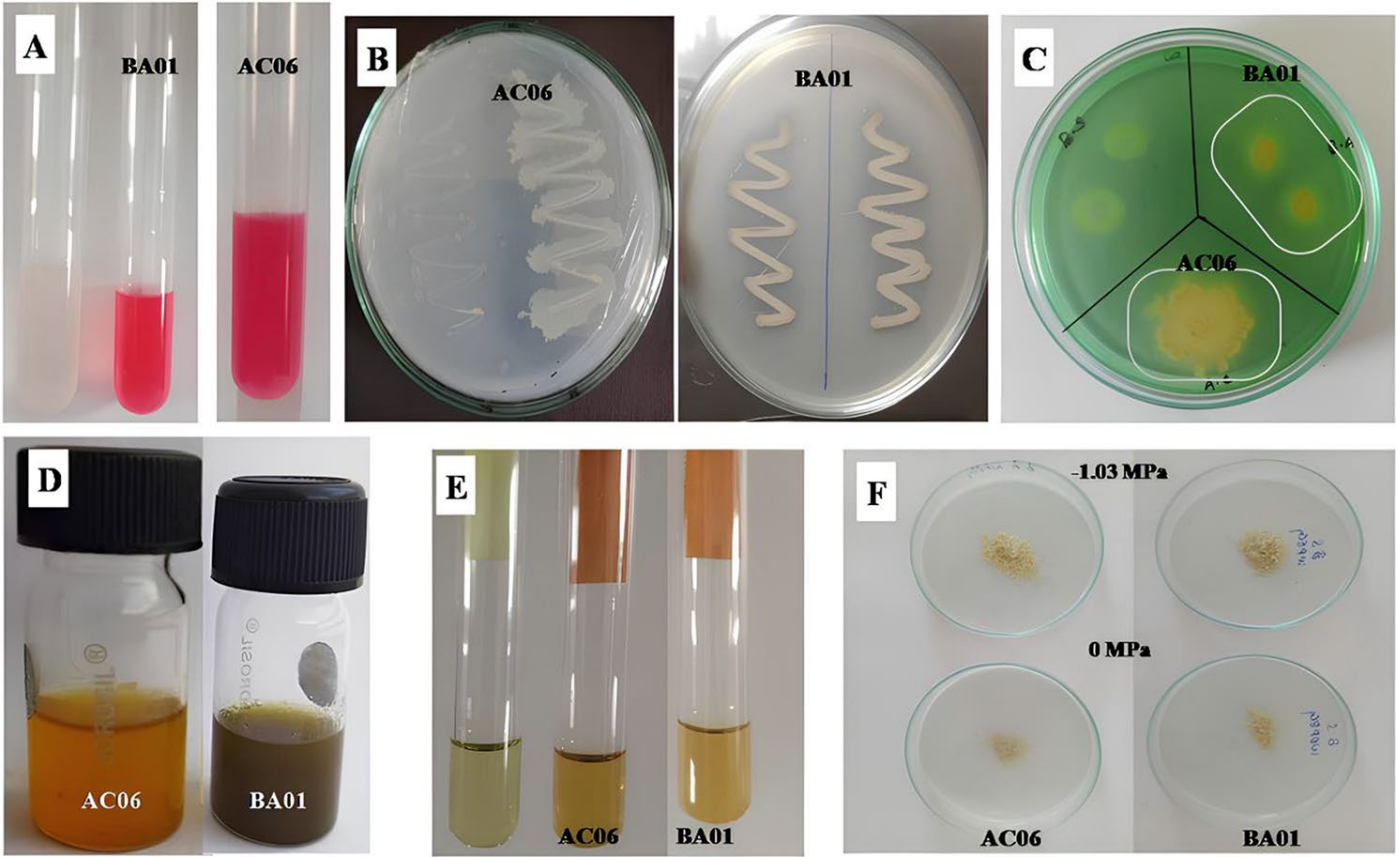
Plant growth promoting analysis of strain *Acinetobacter calcoaceticus* AC06 and *Bacillus amyloliquefaciens* BA01 (**A**) IAA production; (**B**) Phosphate solubilization; (**C**) Siderophore production; (**D**) Ammonia; (**E**) HCN and (**F**) EPS production

A large number of isolates showed siderophore production. Among them, AC06 was the most potent isolate with the most significant siderophore percentage of 84.87 and BA01 with 79.16%. Concerning phosphate solubilization qualitatively, AC06, BA01, SB14, ES12, and SA04 caused the highest value (PSI more than 60). All rhizobacterial isolates have exhibited positive results for NH_3_ except SA04, AC05, ES15, BA06, and SB30. Similarly, the test for HCN was recorded as positive for SA02, SA04, A05, AC06, BA01, BA06 and SB05. Amidst 15 isolates, AC06 and BA01 emerged with exceptional PGP traits (Fig. 1).

### Rhizobacterial growth at varied osmotic pressures

**Fig. 2:**
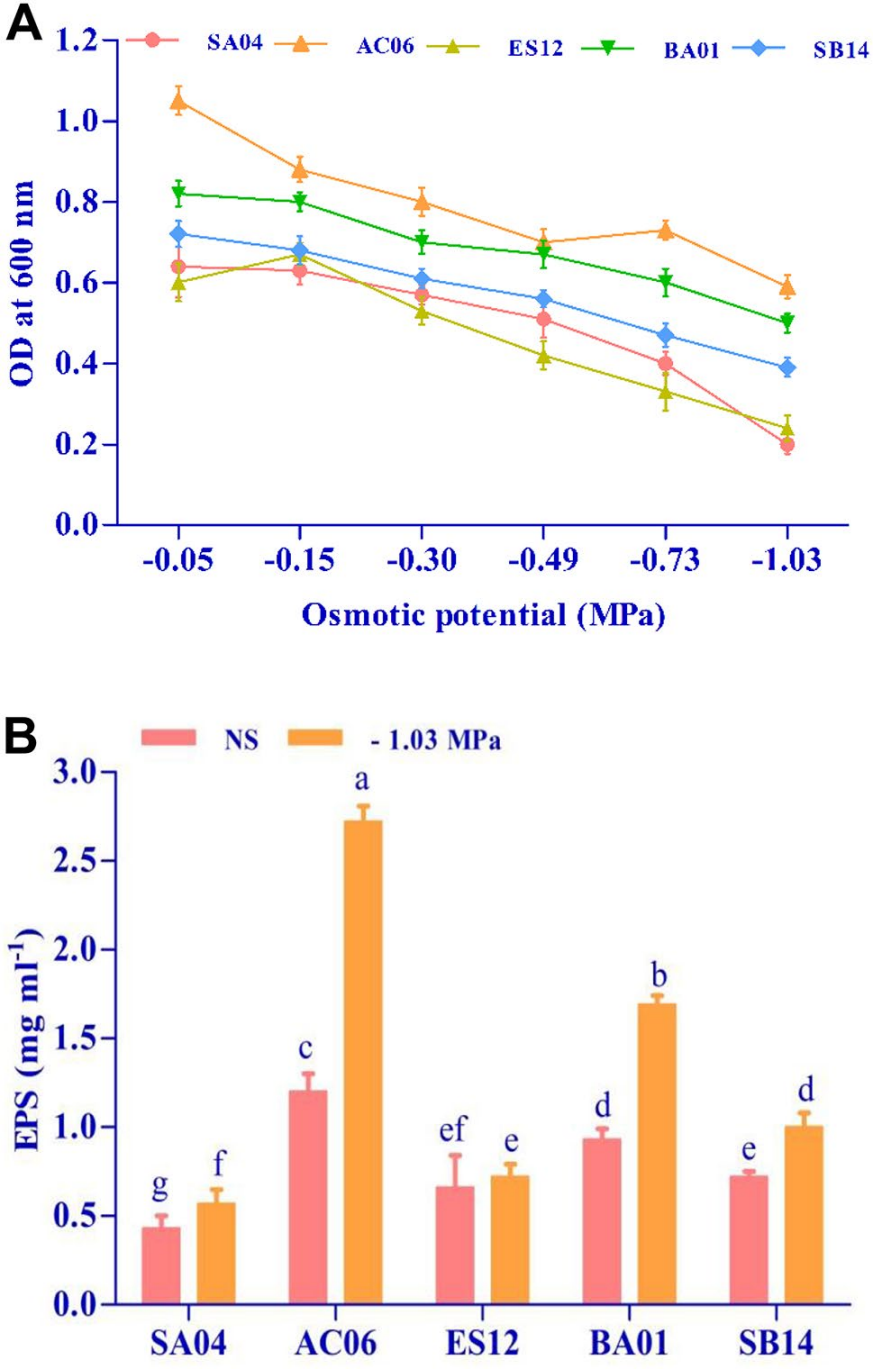
The growth pattern of rhizobacteria under different matric potential (**A**), EPS production (**B**) by other isolates under non-stressed (NS) and drought (-1.03 MPa) conditions. Values are means of three replicates (*n* = 3), and bars denote standard deviation. Different alphabets indicate significant differences between treatments based on Tukey’s *post-hoc* test (*p* ≤ 0.05)

The five most potent isolates were selected based on PGP traits results; SA04, AC06, ES12, BA01, and SB14 were grown in nutrient broth under varied osmotic pressures by PEG 6000 supplementation (Fig. 2A). Of 5 isolates, 2 were highly tolerant, 1 was tolerant, and 2 were highly sensitive. Isolates AC06 and BA01 are grouped as highly tolerant because they have OD > 0.5 at – 1.03 MPa. SB14 was included under the tolerance class as it has OD < 0.4 at – 1.03 MPa. ES12 and SA04 are listed in the susceptible class because the OD decreased below 0.25 at – 1.03 MPa.

#### Exopolysaccharide (EPS) production

Results regarding the production of EPS revealed that osmotic stress had variable effects on all five isolates compared to unstressed conditions, as shown in Fig. 2B. AC06 and BA01 achieved the highest EPS production under unstressed conditions, 1.20 and 0.93 mg ml^− 1^, while at -1.03 MPa, they were significantly raised to 2.72 and 1.69 mg ml^− 1^, respectively. There is no statistical difference in EPS among other isolates under both experimental conditions.

#### Biochemical characteristics and genotyping of osmotolerant PGPR

**Fig. 3:**
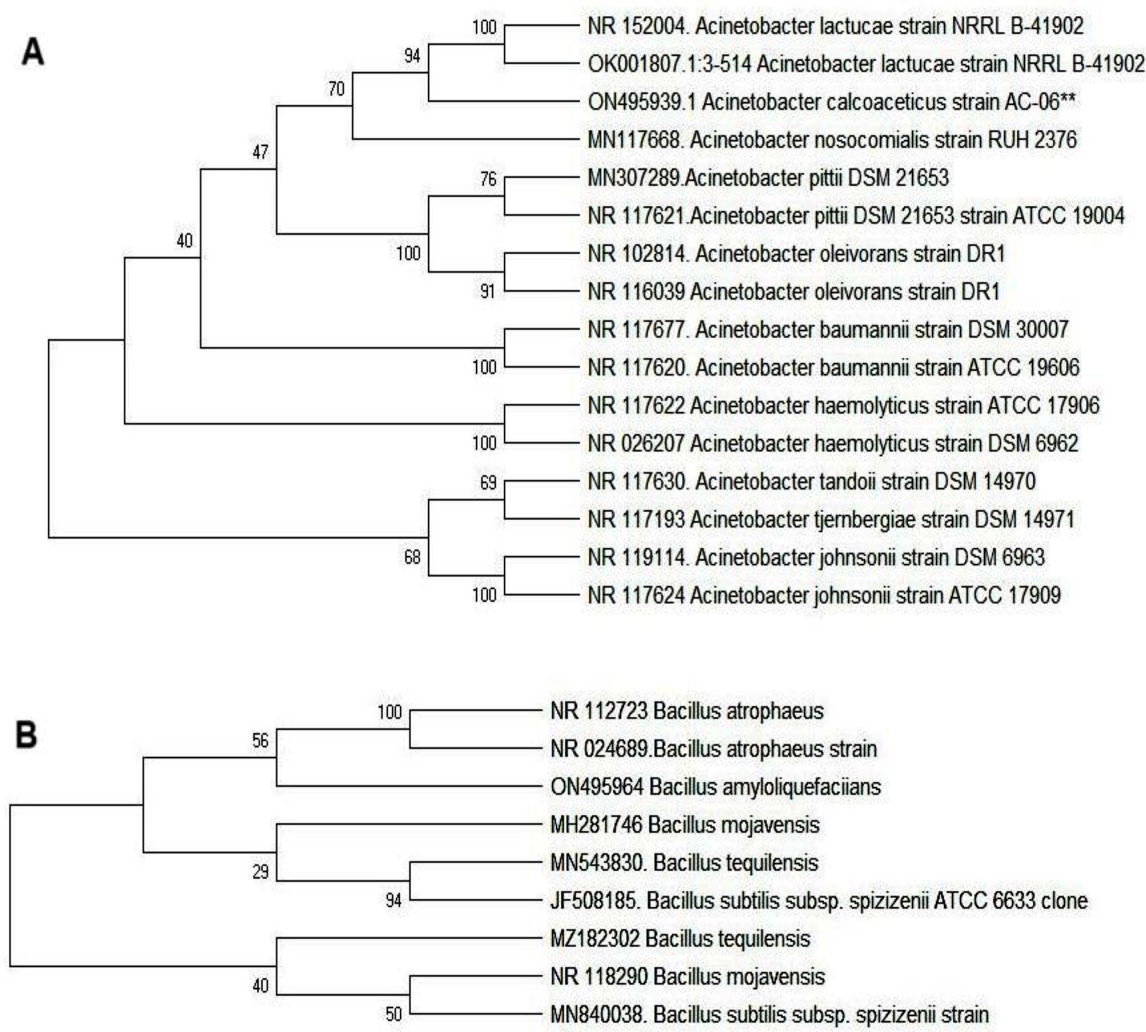
Phylogenetic analysis of (**A**) *Acinetobacter calcoaceticus* (ON495939) (**B**) *Bacillus amyloliquefaciens* (ON495964) revealing evolutionary divergence based on 16S rRNA gene sequences. Neighbor-joining method using MEGA ver.11.0. analyzed distance and clustering

The two most promising strains (AC06 and BA01), showing efficient osmotic tolerance and EPS production, phenotypically resembled *Acinetobacter and Bacillus* spp, respectively (Supplementary Table S1). The strains’ genotypes were identified based on their sequence matched through NCBI-BLASTn. The strain AC06 had a sequence similarity of 99.9% with *Acinetobacter calcoaceticus*, and BA01 displayed a close similarity of 99% to *Bacillus amyloliquefaciens*. The aligned sequences of 16S rRNA were deposited in the GenBank database (NCBI) under the accession number ON495939 for AC01 and ON495964 for the BA01 strain. The ancestral tree was built to designate the strain identification and genotypic characterization. The PGPR biostimulants were thus identified as *A. calcoaceticus* (AC06) and *B. amyloliquefaciens* (BA01) strains (Fig. 3).

#### Osmolytes production of drought-tolerant PGPR biostimulants

**Fig. 4:**
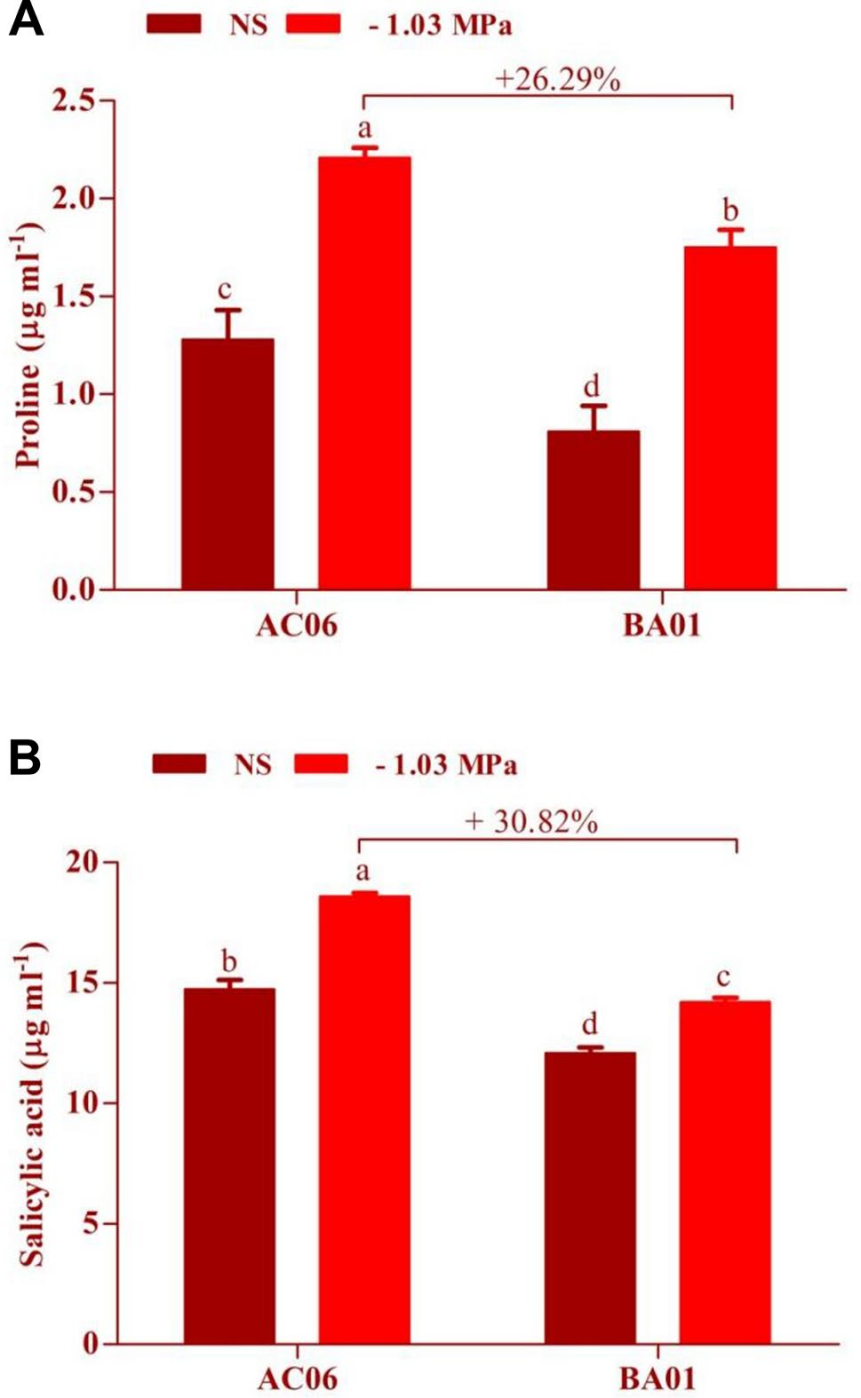
Bar graphs illustrate the osmolyte production of drought-tolerant bacteria under unstressed (NS) and drought (-1.03 MPa) conditions (**A**) Proline and (**B**) Salicylic acid. Values are means of three replicates (*n* = 3), and bars denote standard deviation. Different alphabets indicate significant differences between treatments based on Tukey’s *post-hoc* test (*p* ≤ 0.05)

**Fig. 5:**
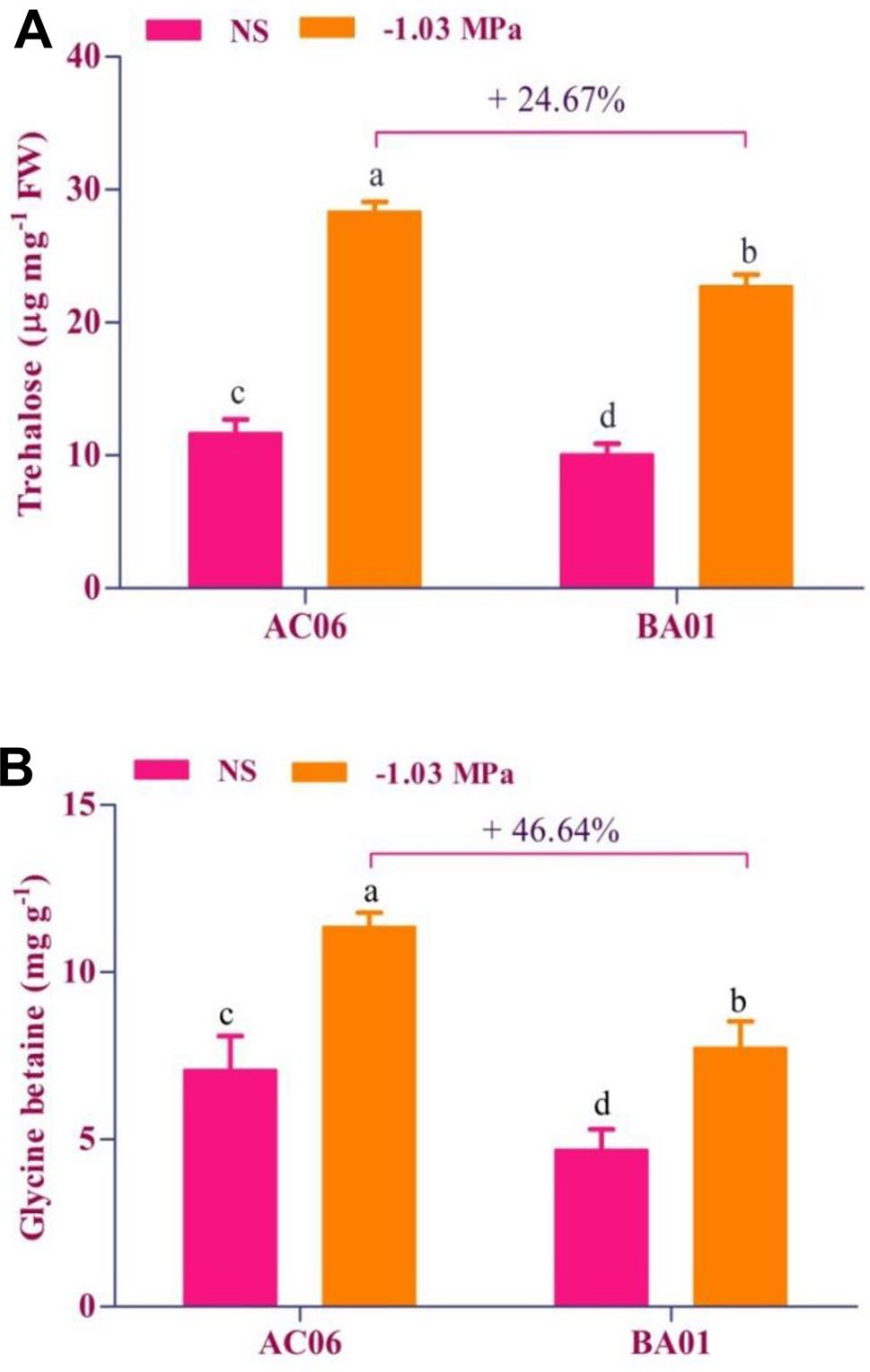
Bar graphs illustrate the osmolyte production of drought-tolerant bacteria under unstressed (NS) and drought (-1.03 MPa) conditions (**A**) Trehalose and (**B**) Glycine betaine. Values are means of three replicates (*n* = 3), and bars denote standard deviation. Different alphabets indicate significant differences between treatments based on Tukey’s *post-hoc* test (*p* ≤ 0.05)

Traits associated with oxidative stress of drought-tolerant PGPR biostimulants such as proline, salicylic acid, trehalose, and glycine betaine were assessed under stressed (− 1.03 MPa) and unstressed conditions, as shown in Figs. 4 and 5. Regarding proline production (Fig. 4A), both strains produced various amounts of proline with the maximum quantities at -1.03 MPa compared to unstressed conditions. Meanwhile, the maximum proline was recorded in AC06 with 26.29% more than BA01 under stress. When AC06 and BA01 were cultured under osmotic stress (-1.03 MPa), the content of salicylic acid was significantly increased by 26.12 and 17.54%, respectively, relative to unstressed control. However, AC06 exhibited 30.82% higher salicylic acid production than BA01 at -1.03 MPa (Fig. 4B).

Trehalose was initially found to be minimum in both strains under unstressed conditions (Fig. 5A). However, it was found to be at peak under stress implementation. The production of trehalose by AC06 under stress was 142.51% greater than that produced without stress and even exceeded the values obtained with strain BA01 by 24.67% at -1.03 MPa. The accumulation of glycine betaine in AC06 was found to be in a similar trend, 46.64% higher than BA01 at maximum osmotic stress, as shown in Fig. 5B. These data demonstrated a positive association between osmotic stress tolerance and osmolyte production in PGPR biostimulants.

### Influence of drought-tolerant PGPR biostimulants on groundnut (*Arachis hypogaea* L.)

Drought tolerant strains *A. calcoaceticus* and *B. amyloliquefaciens* were screened and selected to analyze their impact on the agronomic, physiological, and metabolic quantities of groundnut in a pot experiment under a range of induced drought stress (MD and SD).

#### Effects of PGPR biostimulants on morphological responses of groundnut to drought

Inoculation with PGPR strains as microbial biostimulants significantly increased all evaluated morphological parameters (Table 3) under stress. As expected, groundnut plants’ root length, shoot length, and fresh and dry weight declined significantly with increasing drought stress, with growing under SD having the lowest values. Plants inoculated with AC06 and BA01 under MD and SD recorded a statistical difference in root length and shoot length, approximately 30% and 15% higher than the control. A greater fresh weight (*p* < 0.05) was detected in inoculated plants and subjected to drought stress, regardless of the inoculated strains compared with MD and SD plants. In both drought treatments (MD and SD), analog values of fresh weight were noted. Under 100% FC, the dry mater was not considerably different in both strains. However, under stress applications (MD and SD), dry Matter significantly (*p* < 0.05) increased by 12.15% and 18.31% inoculated with AC06 than the relevant control. Treatment with BA01 did not influence (*p* > 0.05) the dry matter content under MD and SD.

**Table 3:** Effect of PGPR biostimulants (*Acinetobacter calcoaceticus* – AC06 and *Bacillus amyloliquefaciens* – BA01) on plant growth parameters under drought stress

Treatments		Root length (cm)	Shoot length (cm)	Plant fresh weight (g)	Plant dry weight (g)
100% FC	Control (NI)	10.52 ± 0.42^bcd^	26.01 ± 0.21^b^	22.50 ± 0.22^bcd^	4.31 ± 0.19^d^
	AC06	13.52 ± 0.35^a^	30.36 ± 0.42^a^	26.13 ± 0.19^a^	5.69 ± 0.35^a^
	BA01	12.83 ± 0.38^ab^	28.43 ± 0.40^ab^	25.05 ± 0.34^ab^	4.85 ± 0.48^b^
60% FC	MD	9.13 ± 0.22^cd^	21.01 ± 0.41^cd^	20.78 ± 0.17^cd^	4.24 ± 0.16^f^
	AC06 + MD	11.88 ± 0.24^abc^	25.89 ± 0.35^b^	23.33 ± 0.16^abc^	4.77 ± 0.12^c^
	BA01 + MD	9.79 ± 0.50^cd^	23.01 ± 0.41^c^	21.46 ± 0.54^cd^	4.27 ± 0.26^e^
40% FC	SD	8.04 ± 0.20^d^	18.29 ± 0.20^d^	20.05 ± 0.27^d^	3.66 ± 0.61^h^
	AC06 + SD	10.46 ± 0.19^bcd^	22.77 ± 0.54^c^	22.91 ± 0.15^bcd^	4.33 ± 0.50^d^
	BA01 + SD	9.70 ± 0.27^cd^	20.36 ± 0.31^cd^	21.01 ± 0.15^cd^	3.78 ± 0.38^g^

NI: Non-inoculated, MD: Moderate drought, SD: Severe drought

Data represent mean values of triplicates. ± = standard deviation

Different alphabets indicate significant differences between treatments based on Tukey’s test (*p* ≤ 0.05)

Different letters indicate significant differences at *p* ≤ 0.05

#### Effects of PGPR biostimulants on relative water content, electrolyte leakage, and membrane stability index of groundnut under drought stress

**Fig. 6:**
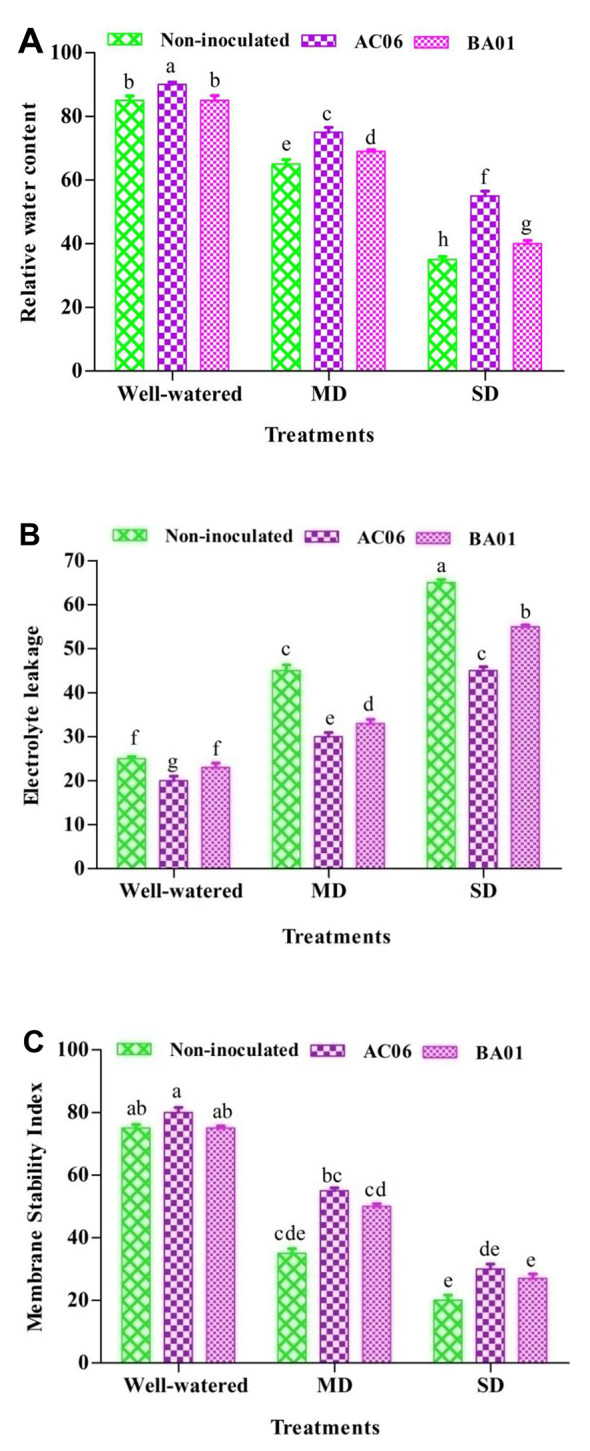
Effect of PGPR biostimulants (*Acinetobacter calcoaceticus* – AC06 and *Bacillus amyloliquefaciens* – BA01) on physiological parameters of groundnut under drought stress (MD: Moderate drought, SD: Severe drought), (**A**) Relative water content; (**B**) Electrolyte leakage and (**C**) Membrane stability index. Values are means of three replicates (*n* = 3), and bars denote standard deviation. Different alphabets indicate significant differences between treatments based on Tukey’s *post-hoc* test (*p* ≤ 0.05)

For relative water content (RWC), a significant (*p* < 0.05) decrease was recorded compared to well-irrigated plants (23.53% reduction in MD and 58.82% reduction in SD). In plants inoculated with AC06 and BA01, RWC was observed significantly (*p* < 0.05) with 15.38 and 6.15% increments, respectively, under MD. Meanwhile, in SD, AC06 notably enhanced the RWC by 57.14% in contrast to stressed plants compared to BA01, as shown in Fig. 6A.

Regarding electrolyte leakage (EL), drought stress caused remarkably high EL% compared to well-watered control (Fig. 6B). However, the values were significantly (*p* < 0.05) decreased in the presence of PGPR strains; the maximum reduction was recorded in plants inoculated with AC06 up to 33.33 and 30.76% under MD and SD compared to respective control. Reduction by BA01 under MD and SD was 26.66 and 15.38%, respectively.

The membrane stability index (MSI) decreased as the levels of drought implication increased. However, PGPR applications increased MSI in all treatment categories, as shown in Fig. 6C. The respective significant increase by PGPR (AC06 and BA01) was from 42.85 to 57.14% under MD and 35 to 50% under SD.

#### Effects of PGPR biostimulants on greenness index, chlorophyll, and carotenoid contents of groundnut under drought stress

**Fig. 7:**
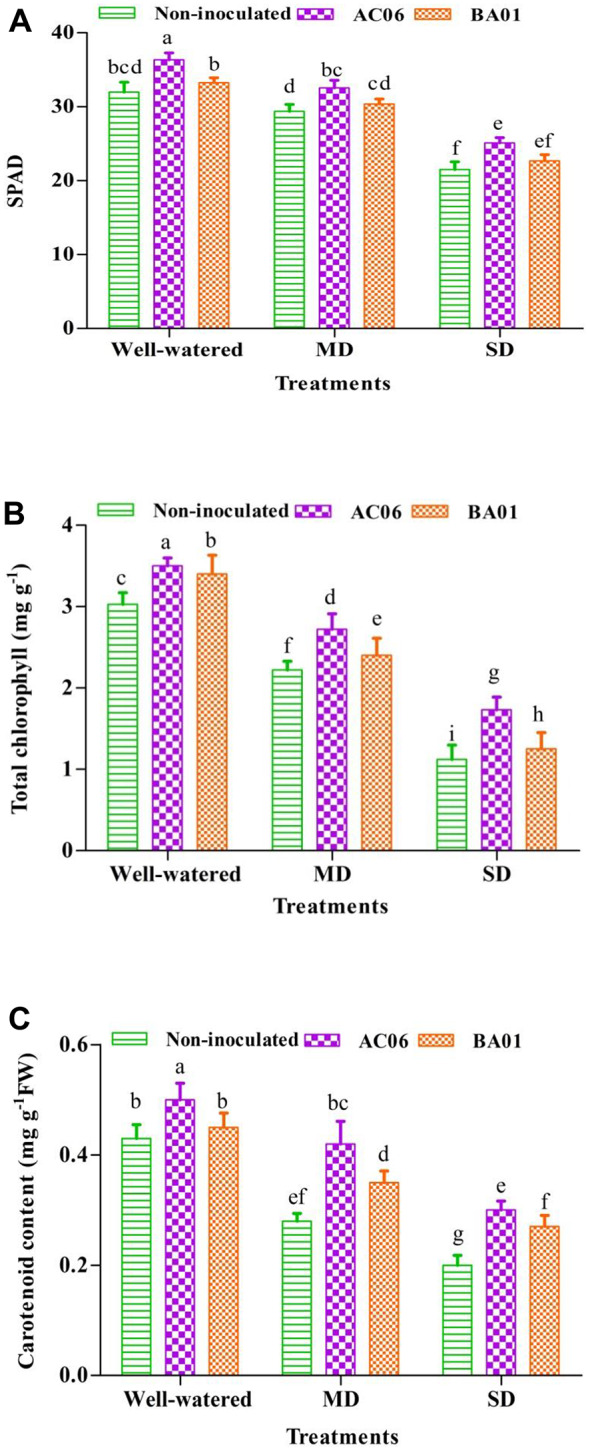
Effect of PGPR biostimulants (*Acinetobacter calcoaceticus* – AC06 and *Bacillus amyloliquefaciens* – BA01) on (**A**) SPAD; (**B**) Total chlorophyll and (**C**) Carotenoid of groundnut under drought stress (MD: Moderate drought, SD: Severe drought. Values are means of three replicates (*n* = 3), and bars denote standard deviation. Different alphabets indicate significant differences between treatments based on Tukey’s *post-hoc* test (*p* ≤ 0.05)

Numeric increments in the greenness index of leaves were statistically (*p* < 0.05) enhanced by 10.68 and 3.29% under MD and 16.58 and 5.29% under SD by AC06 and BA01, respectively, in comparison with corresponding control (Fig. 7A). Similarly, chlorophyll content was also remarkably (*p* < 0.05) higher obtained with AC06 by 22.52 and 54.46% in MD and SD compared to stressed control. Under drought constraints, BA01 did not show any significant difference over specific control plants (Fig. 7B). Carotenoid content improved substantially with inoculation of strains under well-watered and stressful situations (Fig. 7C). Significantly (*p* < 0.05) maximum carotenoid (50%) was observed with AC06 followed by BA01 (25%) in contrast to non-inoculated MD plants. Under severe drought, carotenoid status in groundnut leaves showed a significant (*p* < 0.05) difference between inoculated and non-inoculated plants.

#### Effects of PGPR biostimulants on proline, lipid peroxidation and TSS under drought in groundnut

**Fig. 8:**
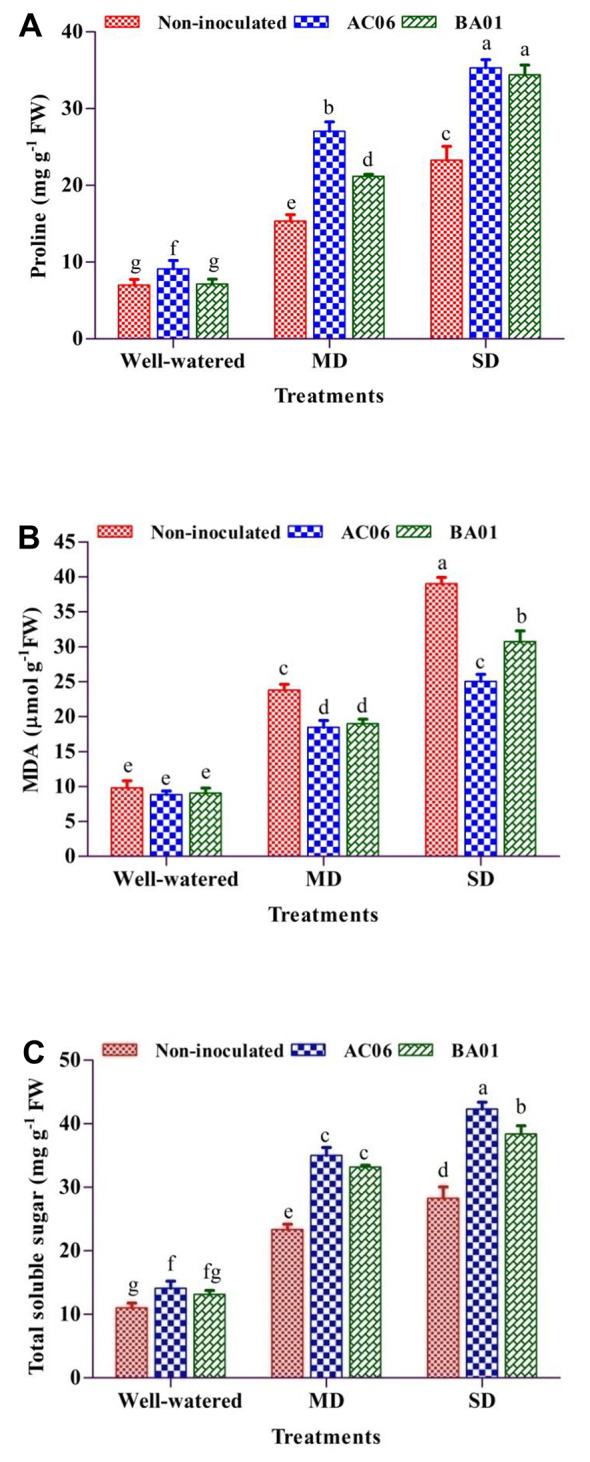
Effect of PGPR biostimulants (*Acinetobacter calcoaceticus* – AC06 and *Bacillus amyloliquefaciens* – BA01) on biochemical characters of groundnut under drought stress (MD: Moderate drought, SD: Severe drought), (**A**) Proline (**B**) Lipid peroxidation (MDA) and (**C**) Total soluble sugar. Values are means of three replicates (*n* = 3), and bars denote standard deviation. Different alphabets indicate significant differences between treatments based on Tukey’s *post-hoc* test (*p* ≤ 0.05)

The proline (non-enzymatic antioxidant) content in groundnut was induced by drought stress (Fig. 8A). The drought-stressed plants (MD and SD) showed 118.80 and 231.77% increments in proline compared to well-watered control. Rhizobacterial strains significantly (*p* < 0.05) enhanced the proline concentration compared to un-inoculated under drought. Proline was maximally induced by inoculation of AC06, representing 76.04% more MD stress than BA01. However, under SD, both AC06 and BA01 significantly (*p* < 0.05) manifested the proline concentration by 50% than the respective control.

As shown in Fig. 8B, MD and SD induced overproduction of ROS, resulting in lipid peroxidation (represented by MDA accumulation). MDA was not significantly affected (*p* > 0.05) between treatments in well-watered plants. MDA was notably increased in stressed plants (MA and SD) by 143.20 and 298.36% respectively. Under MD, the rhizobacterial applications induced a 20% reduction in MDA compared to stressed plants. On the other hand, bacterial inoculation decreased (*p* < 0.05) leaves’ MDA content by 35.82% in the case of AC06 and 21.21% by BA01 in SD stress. AC06 demonstrated better results compared to BA01.

The tremendous increase in TSS has been documented in drought-exposed plants, i.e., 111.97% and 156.71% in MD and SD. Inoculation of AC06 imparted enhanced results of TSS by 50% and 49.55%, followed by BA01 with 41.99% and 35.53% under MD and SD regimes (Fig. 8C).

#### Effects of PGPR biostimulants on stress markers under drought in groundnut

**Fig. 9:**
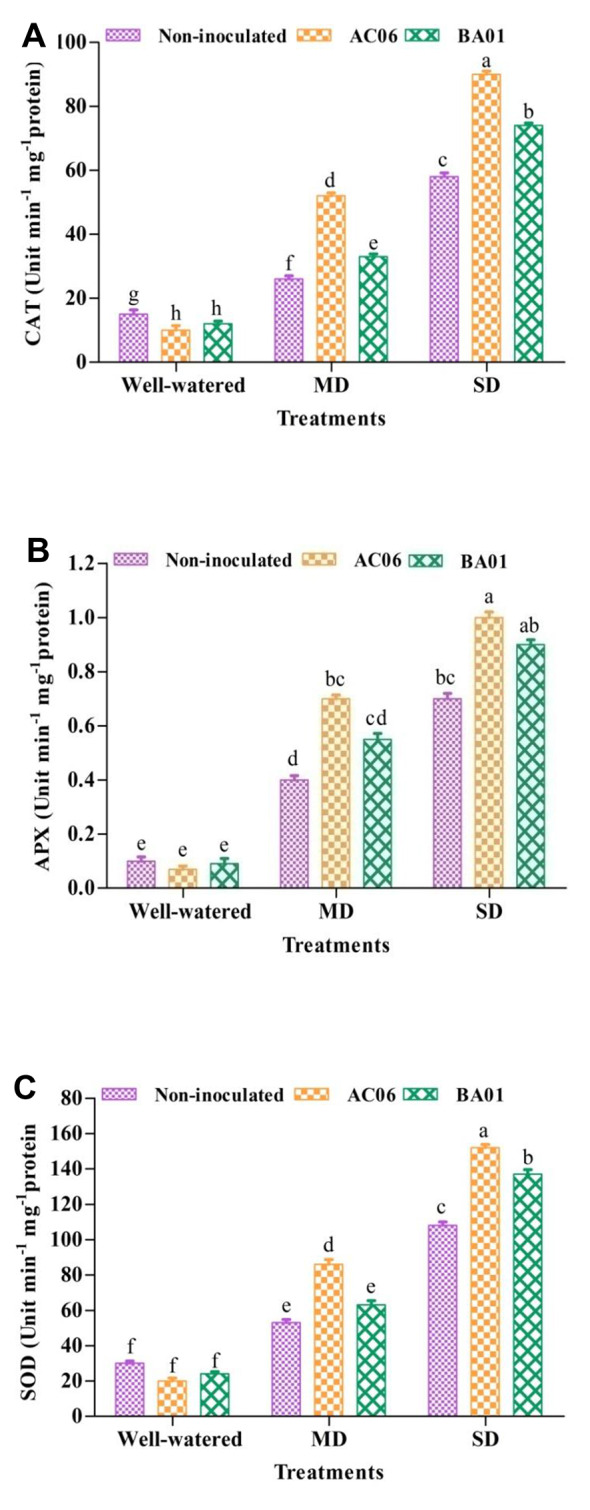
Effect of PGPR biostimulants (*Acinetobacter calcoaceticus* – AC06 and *Bacillus amyloliquefaciens* – BA01) on antioxidant enzyme activities under drought stress (MD: Moderate drought, SD: Severe drought), (**A**) Catalase; (**B**) Ascorbate peroxidase and (**C**) Superoxide dismutase. Values are means of three replicates (*n* = 3), and bars denote standard deviation. Different alphabets indicate significant differences between treatments based on Tukey’s *post-hoc* test (*p* ≤ 0.05)

In addition to non-enzymatic antioxidant (proline), PGPR biostimulants led to statistically (*p* < 0.05) significant impact on the activity of CAT, APX, and SOD (enzymatic antioxidant stress markers) (Fig. 9). A substantial difference in the antioxidant enzyme activities was observed between inoculated and un-inoculated treatments under drought stress. Statistically high (*p* < 0.05) CAT enzyme activity under MD was recorded in plants inoculated by AC06 (100%) followed by BA01 (26.92%) in contrast to control. Similarly, the activity increased under severe drought (AC06-55.17% and BA01-27.57%) compared to stressed control (Fig. 9A).

The increase in APX was 75 and 37.5% by AC06 and BA01 compared to the un-inoculated MD control. In contrast, in SD, the enzymatic activity of AC06 was significantly (*p* < 0.05) more than BA01 by 14.29% (Fig. 9B). SOD production was also augmented for combating drought stress tolerance in groundnut. Application of AC06 and BA01 statistically (*p* < 0.05) increased SOD activity up to 62.26 and 18.87% in MD and 40.74 and 26.85% in SD compared to the respective control (Fig. 9C).

#### Pearson’s correlation coefficient analysis

**Fig. 10:**
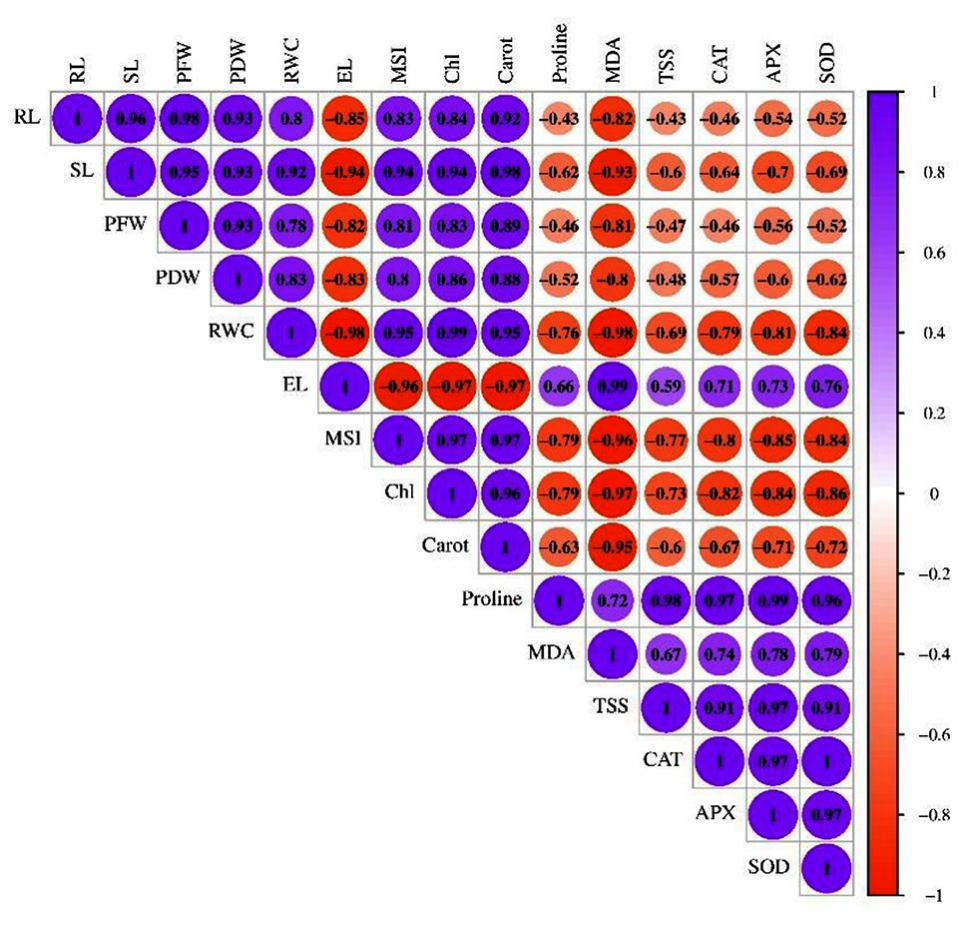
Pearson’s correlation between morphological, physiological and biochemical attributes of groundnut treated with microbial biostimulants under drought stress. RL, root length; SL, shoot length; PFW, plant fresh weight; PDW, plant dry weight; RWC, relative water content; EL, electrolyte leakage; MSI, membrane stability index; Chl, chlorophyll; Carot, carotenoid; MDA, lipid peroxidation; TSS, total soluble sugar; CAT, catalase; APX, ascorbate peroxidase and SOD, superoxide dismutase

Pearson’s correlation matrix showed a strong significant positive correlation among RL, SL, PFW, PDW, RWC, MSI, Chl and Carotenoid (*p* < 0.01) as well as between Proline, MDA, TSS, CAT, APX and SOD (*p* < 0.01) (Fig. 10). In addition, a strong negative correlation of EL and MDA with RL, SL, PFW, PDW, RWC, MSI, Chl, and Carotenoid (*p* < 0.05) was observed. Also, negative correlation between RL, SL, PFW, PDW, RWC, MSI, Chl, and Carotenoid with Proline, MDA, TSS, CAT, APX and SOD was perceived. While a strong positive correlation was seen between EL and MDA (*p* < 0.01), and a positive correlation of EL and MDA with Proline, TSS, CAT, APX and SOD (*p* < 0.01) was observed.

#### Effect of PGPR biostimulants on the rhizospheric bacterial population of groundnut

The application of the PGPR significantly enhanced bacterial colonization under standard and drought-stressed plants (Supplementary Table S2). The maximum increase in the bacterial count was 6.46 and 6.18 log CFUg^− 1^soil under the application of AC06 and BA01 in non-stressed conditions. However, drought stress slightly affected the colonization in AC06 treated plants with a 14 and 25% reduction, while BA01 treated plants showed 22 and 36% reduced bacterial count under MD and SD.

## Discussion

Drought stress is a recurring climatic issue to global agro-sustainability since its alarming intensity impairs plant growth, hinders nutrient absorption, reduces photosynthesis, alters metabolic processes, and ends in oxidative stress [56–58]. The application of microbial biostimulants confers resistance and tolerance against drought stress *via* RIDER (Rhizobacteria-induced drought endurance and resilience) mechanisms as well as plays an integral role in resolving food security issues and nutrient availability in crops [59, 60]. Isolation and screening of rhizobacteria for desired functional traits are expected to provide the most effective strain of PGPR for plant stress management. Formerly, two different rhizobacteria were characterized as putative PGPR biostimulants [23] and evaluated for plant growth promotion in groundnuts under pot and field trials. With prospects of designing PGPR-based microbial biostimulants with improved drought-resistance strains, the present investigation aimed to isolate and characterize osmo-adaptive features of rhizobacteria and induction of growth potential in groundnut under osmotic stress. We performed in vitro screening of 15 indigenous rhizobacteria isolated from arid and semi-arid regions for their highest drought-tolerance activity along with PGP activities. Our findings agree with reports of previous investigations on alleviating drought stress by using PGPR biostimulants [61, 62].

Many bacterial genera exhibit plant growth- promoting traits in different crops [63, 64]. The drought-tolerant PGPR ameliorates drought stress through PGP attributes [65]. Production of rhizobacterial IAA is a direct mechanism to enhance plant development, which was efficiently produced by the selected bacteria of the present work. Jayakumar et al. [66] mentioned the central role of IAA in stimulating plant cell growth and division, thereby improving root absorptive surface area [67]. Phosphate is a primary macronutrient that increases plant immunity, protecting it from drought stress [63]. A remarkable percentage of isolates (73.33%) were positive for phosphate solubilization. Danish and Zafarul-Hye [68] and Kour et al. [69] reported similar results on siderophore, HCN, and ammonia production.

Siderophore production is one of the microbes’ essential survival strategies because they chelate Fe^3+^, and improves its solubility and uptake, assisting plant growth under iron-limiting conditions [67]. Numerous strains of PGPR produced HCN as antibiosis activity, an indirect mechanism for plant growth promotion. Several researchers have documented the generation of ammonia by drought–tolerant soil bacteria induced by polyethylene glycol [70]. Different osmotic pressures generated through PEG 6000 examined the best five isolates for their growth pattern under PEG that reduced water availability and thus mimicked drought stress [71]. Two (AC06 and BA01) out of five analyzed isolates tolerated an exceptionally higher water potential of – 1.03 MPa (30% PEG), suggesting their natural adaptation feature to drought.

EPS production has been suggested as a defence response triggered by stress. Therefore, it is an essential criterion for plant growth promotion under drought stress [72]. EPS plays a significant role in physiological adaptation, enabling bacterial cells to survive under water-limiting conditions because it is hygroscopic and possesses unique water-retention and cementing properties, which imparts protection to bacteria and host plants against desiccation through improved soil aggregation [73]. All five isolates produced EPS under stress (-1.03 MPa) conditions compared to non-stress conditions; however, isolates AC06 and BA01 produced more EPS. The tolerance of these bacterial strains to low osmotic levels was probably because of EPS production in semi-arid habitats. These results were in line with previous studies where high EPS secretion of *Pseudomonas fluorescens* DR7, *Bacillus* sp., and *Enterobacter* was deemed responsible for bacterial attachment, plant-bacteria interaction, and root colonization under harsh drought environment [72, 74, 75]. In vitro PGP traits, drought resilience test, and EPS activity of selected bacterial isolates outlined that AC06 and BA01 were exceptionally remarkable in the current analysis. Genotypic identification based on 16S rRNA gene homology depicted that these selected drought-tolerant strains (AC06 and BA01) belong to *A. calcoaceticus* and *B. amyloliquefaciens*, respectively.

Microbial biostimulants produce several osmolytes to adapt to stressful environments. A better understanding of the osmo-adaptive mechanisms of the AC06 and BA01 strains would aid in improving the interaction of these bacteria with plants under water scarcity. In this sense, the production of compatible osmolytes by PGPR, such as proline, trehalose and glycine betaine, along with the salicylic acid, was measured. Notably, these strains produced osmolytes, suggesting a strong association between osmotic stress and survival in low water potential. Proline and salicylic acid by PGPR can be linked to the degree of drought tolerance and its endurance in soil [74, 76]. Ashry et al. [34] reported the maximum production of proline and SA by DS4 under the harsh conditions of PEG. In literature, an exceptional behavior of high trehalose production by *Azosprillum* strain Az19 was observed under mild stress [33]. Vilchez et al. [77] also revealed the correlation between microbial trehalose and its stability under drought. Vasconcellos et al. [43] predicted glycine betaine synthesis acted as an osmoprotectant, maintaining the cell turgor pressure for bacterial survival.

Variation in the patterns of growth enhancement has been observed when plants are treated with microbial biostimulants under osmotic stress. The analyzed data demonstrated the ability of *A.calcoaceticus* AC06 and *B. amyloliquefaciens* BA01 showing significant tolerant to drought in groundnut by measuring growth indices, photosynthetic pigments, RWC, EL, MSI, MDA, TSS, proline and ROS-scavenging enzymes. At exposure to drought, plants exhibited a halt in growth parameters, further ameliorated by inoculation of biostimulants. The present study’s findings also agreed with the research conducted by Kumar et al. [78], where root length, shoot length, and plant fresh and dry weight were more pronounced in groundnuts by drought mitigating the PGPR consortium. Inoculation of *A. calcoaceticus* EU-LRNA-72 in foxtail millet showed drought mitigation and physiological growth by osmolyte adjustments [79]. Plants with AC06 and BA01 inoculation showed an essential increase in RWC and MSI but a reduction in EL over plants without inoculation under MD and SD. Comparable results were reported in alfalfa treated with PGPR QST713 [80] and wheat treated with FAP5 strain under drought [81]. This may suggest that PGPR under stress can potentially improve plant water content, thereby regulating membrane stability and electrolyte leakage by altering hydraulic conductivity [2].

Inhibition of photosynthetic process leads to the generating of reactive oxygen species [82]. This action likely interferes with the pyrrole biosynthesis pathway, which is indispensable for chlorophyll formation [83]. But in the prevailing research, PGPR-based microbial biostimulants AC06 and BA01 significantly increased the chlorophyll content in groundnuts as compared to stressed plants, thus improving the light-harvesting capacity, enhancing the supply of CO_2_ and reducing the formation of reactive oxygen species in plants. This fact is also acknowledged by the study of Ansari et al. [81] and Khalilpour et al. [84], ideally proving the ability of PGPR strains on the photosynthetic machinery under drought. Overall, the findings above indicated the favourable effect of microbial biostimulants may be due to a reduction in stress, induced changes in the structure of chloroplast, and repair of photosynthetic enzymes [68], supporting its positive influence on plant growth and physiological parameters [85].

Proline and TSS are the major compatible osmolytes accumulated in plants under drought stress, and their link confers drought tolerance [86]. Correspondingly, enhanced accumulation of proline and TSS in groundnut leaves was noted upon priming with AC06 and BA01 under MD and SD. Besides ROS scavenging, proline is an essential osmolyte that maintains the cell membrane’s integrity and structure. Higher TSS (47 mg/g FW) by hydrolysis of starch provides osmotic adjustment under drought [87, 88]. In the present analysis, a reduced level of MDA was detected under drought-stressed conditions in contrast to non-inoculated drought plants. These results were correlated with the earlier findings [89, 90], which showed tolerance by microbial biostimulants against drought stress was positively correlated with reduced MDA levels. Mellidou et al. [91] equivalently confirmed a reduction in MDA content, implying that strain inoculation possibly mimicked the impact of mild oxidative stress, which could prime the plant toward stress application.

An increase in antioxidant enzyme activity is vital in plant stress resistance. Plants have a well-organized defense mechanism for scavenging ROS generated during drought stress [92, 93]. The enzyme superoxide dismutase (SOD) releases hydrogen peroxide by dismutation of superoxide radicals [94]. This hydrogen peroxide damages the cell membrane, and the catalase (CAT) enzyme further induces the reduction of hydrogen peroxide into water and molecular oxygen [95, 96]. We described the results with groundnut plants displaying higher activities of antioxidant enzymes (SOD, CAT, and APX) under drought stress. Sukweenadhi et al. [97], Sharma et al. [98], Vaishnav et al. [99], and Khan and Bano [91] achieved similar patterns of findings. According to our data, elevated activities of the antioxidant enzymes CAT, APX, and SOD resulted in enhanced drought tolerance in groundnut, and *A. calcoaceticus* (AC06) and *B*. *amyloliquefaciens* (BA01) have been found to mediate more excellent drought resistance by manipulating the ROS-scavenging enzymes and can be exploited efficiently under hostile environments. The activity of antioxidants is imperative during acute drought stress and interferes with recovery from water deprivation and dehydration resuscitation [100]. Substantial studies have indicated the drought stress tolerance and inoculation-induced ROS-scavenging enzymes in plants [85, 101, 102]. PGPRs stimulate the host plant’s defense mechanism and boost its antioxidant scavenging system [88]. Bacterial root colonization is responsible for the enhanced activity of PGPR for plant growth promotion [103, 104]. PGPR-treated plants exhibited high rhizospheric colonization, whereas the extent of colonization was reduced under drought stress. However, the bacterial population CFU count showed the PGPR’s ability to survive and colonize even during drought. Ahmed et al. [70] reported the enhanced microbial population by applying PGPR, favoring the presence of inoculated bacteria for longer under water deficit conditions. PGPR colonization may have triggered the plant’s physiological mechanism, which is its development under drought. Therefore, the results presented here support the hypothesis that the osmolyte-producing rhizobacteria with plant growth-promoting attributes can contribute to the drought habitat adaptation of oil-yielding plants like groundnuts.

## Conclusion

Drought tolerant, osmolyte, and EPS-producing rhizobacteria associated with groundnut could counteract drought stress, as indicated by accelerated growth variables, physiological redox status, and activity of stress markers. We suggest that osmotolerant PGPR belonging to genera *Acinetobacter* and *Bacillus* could help develop microbial biostimulants for abiotic stress management in plants. To our knowledge, ours is the first report describing the osmolyte production of *Acinetobacter* sp. AC06 and *Bacillus* sp. BA01, especially in oil-seed crops under extreme drought. Despite their potential, no commercial products based on these species are available. As evidence, the present research suggests that microbial biostimulants products will probably be launched in the future. Henceforth, a mechanistic framework explaining the mode of action of microbial biostimulants will be taken to devise a roadmap for biostimulant-based strategies for sustainable food security and resource use efficiency.

### Electronic supplementary material

Below is the link to the electronic supplementary material.

Supplementary Material 1

## Data Availability

The aligned sequences of 16S rRNA are available in the GenBank database (NCBI) under the accession number ON495939 for AC01 and ON495964 for the BA01 strain.

## Abbreviations

AC06: *Acinetobacter calcoaceticus*
APX: Ascorbate Peroxidase
BA01: *Bacillus amyloliquefaciens*
CAT: Catalase
CFU: Colony Forming Unit
EL: Electrolyte Leakage
EPS: Exopolysaccharides
FC: Field Capacity
HCN: Hydrogen cyanide
IAA: Indole Acetic Acid
MD: Mild Drought
MDA: Malondialdehyde
MSI: Membrane Stability Index
NB: Nutrient Broth
NH_3_: Ammonia
NI: Non Inoculated
PEG: Polyethylene Glycol
PGPR: Plant Growth Promoting Rhizobacteria
PSI: Phosphate Solubilization Index
ROS: Reactive Oxygen Species
RWC: Relative Water Content
SA: Salicylic Acid
SD: Severe Drought
SOD: Superoxide Dismutase
TBA: Thiobarbituric Acid
TCA: Trichloroacetic Acid
TSA: Tryptic Soy Agar
TSS: Total Soluble Sugar
