# Supplementary material for: Osmolyte-producing microbial biostimulants regulate the growth of Arachis hypogaea L. under drought stress
Source: BMC Microbiol. 2024 May 15;24:165. doi: 10.1186/s12866-024-03320-6 (PMC11094965; doi:10.1186/s12866-024-03320-6)
Supplement: Supplementary file 1 — Supplementary Material 1 [file 12866_2024_3320_MOESM1_ESM.docx]

**Table S1**

Morphological and biochemical characteristics of *Acinetobacter* *calcoaceticus* AC06 and *Bacillus* *amyloliquefaciens* BA01

| **S.No** | **Characteristics** | **Result** | |
| --- | --- | --- | --- |
|  |  | **AC06** | **BA01** |
|  | Colour | White | Cream white |
|  | Cell shape | Cocci | Bacilli |
|  | Colony margin | Undulate | Lobate |
|  | Surface appearance | Rough | Smooth |
|  | Gram stain | - | + |
|  | Indole test | + | - |
|  | MR test | + | - |
|  | VP test | + | + |
|  | Citrate utilization | + | + |
|  | Oxidase | **+** | + |
|  | Catalase | + | + |
|  | Starch hydrolysis | - | + |

(+) Positive; (-) Negative

**Table S2**

**Rhizospheric bacterial population after inoculation of PGPR biostimulants under drought**

| **S.No** | **Treatments** | **log CFU g^-1^ soil** |
| --- | --- | --- |
|  | Control (NI) | 3.66 ± 0.05 |
|  | AC06 | 6.46 ± 0.07 |
|  | BA01 | 6.18 ± 0.22 |
|  | MD | 2.98 ± 0.10 |
|  | AC06 + MD | 5.52 ± 0.15 |
|  | BA01 + MD | 4.76 ± 0.27 |
|  | SD | 2.05 ± 0.08 |
|  | AC06 + SD | 4.81 ± 0.04 |
|  | BA01 + SD | 3.92 ± 0.11 |

Data are represented by mean± standard deviation.
